# Independent validation of lung adenocarcinoma prognostic risk scores incorporating cholesterol and estrogen metabolism related transcriptional biomarkers

**DOI:** 10.1038/s41598-025-22140-w

**Published:** 2025-10-31

**Authors:** Qian Zhu, Yuemei Zhang, Jian Ma, Yongjia Li, Hongya Liu, Zhongwen Gong, Ming Du, Xuemei Lian

**Affiliations:** 1 Center for Lipid Research, Key Laboratory of Molecular Biology for Infectious Diseases (Ministry of Education), Chongqing Medical University, No. 1 Yixueyuan Rd, Yuzhong District, Chongqing, 400016 China; 2 Department of Nutrition and Food Hygiene, College of Public Health, Chongqing Medical University, Chongqing, 400016 China; 3 Clinical Medical Research Center, Meteorological Medical Research Center, Panzhihua Central Hospital, Sichuan, 617000 China; 4 Department of Cardiothoracic Surgery, The Second Affiliated Hospital, Chongqing Medical University, No. 74 Linjiang Rd, Yuzhong District, Chongqing, 400010 China

**Keywords:** Cholesterol, Estrogen, Metabolic scores, Lung adenocarcinoma prognosis, Immune response, Biomarkers, Cancer genomics, Oncology, Cancer genomics, Lung cancer, Tumour biomarkers

## Abstract

We investigated the prognostic significance of cholesterol metabolism-related genes (CMRGs) and estrogen metabolism-related genes (EMRGs) in lung adenocarcinoma (LUAD). Transcriptomic and clinical data from the cancer genome atlas (TCGA) and gene expression omnibus (GEO) databases were analyzed. Iterative sure independence screening and least absolute shrinkage and selection operator (ISIS-LASSO) Cox regression identified prognostic CMRGs and EMRGs. Two risk scores—Cholescore and Estrogenscore—were constructed and validated. High Cholescore was associated with poor overall survival (OS), reduced immune infiltration, low immune checkpoint expression, and high tumor purity, suggesting an immunosuppressive tumor microenvironment. Estrogenscore also independently predicted LUAD prognosis. Mediation analysis revealed that estrogen-related pathways partially mediated the impact of cholesterol metabolism on prognosis. A significant interaction between Cholescore and Estrogenscore was identified, and patients with both high scores had the worst OS and lowest predicted immunotherapy benefit. Combining the two scores significantly improved the area under the curve (AUC) for 1–5 years OS prediction. These findings suggest that integrating cholesterol and estrogen metabolism signatures can improve LUAD prognostic stratification and provide molecular insights into tumor–immune interactions and immunotherapy response prediction.

**Supplementary Information:**

The online version contains supplementary material available at 10.1038/s41598-025-22140-w.

## Introduction

Lung cancer is the leading cause of cancer-related deaths, accounting for more deaths than breast cancer, prostate cancer and colorectal cancer combined^1^. As low-dose computed tomography screening programs for lung cancer becomes more widely adopted as a core element of clinical practice, it’s possible to screen out greater percentage of patients at an early stage, and new diagnosis and treatment strategies together with early surgery could be applied to improve the prognosis of patients with lung cancer^2,3^. The significant heterogeneity of clinical prognosis in patients with similar clinical characteristics suggests the importance of exploring novel molecular mechanisms and integrating prognostic related genes with traditional clinical parameters to predicting lung cancer prognosis and improving survival.

Reprogramming of cholesterol metabolism, including up-regulation of intracellular cholesterol levels and abnormal accumulation of metabolites, occurs during the development of tumors^4^. Emerging evidence has connected cholesterol metabolism with lung cancer morbidity and mortality, and dysregulated cholesterol homeostasis has been found in human lung cancer tissues and tumor-associated macrophages^5,6^. What’s more, the effects of serum total cholesterol and intracellular cholesterol on the development of lung cancer are not parallel. Cohort studies suggested that serum high cholesterol is inversely associated with lung cancer risk^7^, but lipidomic evidence indicated increased accumulation of free cholesterol and cholesterol esters in primary human lung tumors^8^. Moreover, studies gradually emphasized the coupling role of intracellular cholesterol in pulmonary immunity^9^. Impaired cholesterol efflux mechanism leads to abnormal aggregation of macrophage foam cells and alveolar proteinosis^10,11^. A new study^12^ has shown that the deficiency of efflux pathway in epithelial progenitor cells leads to the accumulation of cholesterol in the tumor microenvironment, limits the infiltration of myeloid cells, and promotes the progression of lung cancer. These evidences may suggest that defining cholesterol and immune metabolism in the local lung microenvironment may be more important than simply measuring serum cholesterol level.

Moreover, since cholesterol is a synthetic precursor of steroid hormones, the interactions between cholesterol metabolism and estrogen have been recognized to regulate the progression of multiple cancer types. Intracellular cholesterol is closely linked to the expression and activation of estrogen related receptor α in breast cancer^13,14^. Estrogen dose-dependently increased the expression of SREBP2, HMGCR and LDLR and inhibits PCSK9-Mediated LDLR degradation in HepG2 cells^15,16^. From one hand, the importance of estrogen signaling in lung carcinogenesis has largely been demonstrated. Researchers have found the significant increases of intertumoral estradiol concentrations^17^, and M2 macrophages, Treg cells, and MDSC cells can be stimulated by physiologic or locally produced estrogen, which could drive cell proliferation, promote obstruction, and dampen antitumor immunity^18^. While, as far as we know, studies systematically incorporating pulmonary cholesterol and estrogen metabolism together in the progression of lung cancer are still limited.

In this study, we integrated the expression profiles of cholesterol metabolism-related genes (CMRGs) in lung adenocarcinoma (LUAD) patients from the cancer genome atlas (TCGA), identified key gene probes that can predict the prognosis of LUAD, and constructed prognostic risk score. Robust validation was performed in both training and independent validation populations, and LUAD patients with high cholesterol scores had a worse prognosis. Patients with different cholesterol prognostic risk score have different functional characteristics, immunophenotypes and immunotherapy response possibility. Finally, we further characterized the functional crosstalk of tissue cholesterol and estrogen metabolism with lung cancer immunity in conjunction with estrogen metabolism prognostic score.

## Results

### Patient characteristics

408 LUAD samples from TCGA cohort and 559 CMRGs were included in the discovery analysis. Among 408 participants from TCGA-LUAD cohort (mean age 65.01 years, 52.5% female), 223 patients (54.7%) were in clinical stage I and 146 patients (35.8%) died. The median overall survival (OS) was 32.04 months. Individual characteristics and clinical information of the validation cohort from the Gene Expression Omnibus (GEO) datasets were described in Table 1.

**Table 1:** Demographic and clinical descriptions of LUAD patients with GEO and TCGA databases.

Variables	TCGA	GSE26939	GSE30219	GSE31210	GSE3141^a^	GSE37745	GSE50081	GSE72094
	(N = 408)	(N = 116)	(N = 85)	(N = 204)	(N = 58)	(N = 106)	(N = 127)	(N = 442)
Age	65.01 ± 10.22	64.04 ± 10.88	61.49 ± 9.28	59.60 ± 7.50	–	62.94 ± 9.22	68.73 ± 9.71	69.30 ± 9.33
Gender, *n* (%)								
Female	214 (52.5)	51 (44.0)	19 (22.4)	109 (53.4)	–	60 (56.6)	62 (48.8)	240 (54.3)
Male	194 (47.5)	49 (42.2)	66 (77.6)	95 (46.6)	–	46 (43.4)	65 (51.2)	202 (45.7)
NA	0	16 (13.8)		0		0	0	
Clinical stage, *n* (%)								
I	223 (54.7)	62 (53.4)	–	109 (53.4)	–	70 (66.0)	92 (72.4)	265 (60.0)
II	94 (23.1)	19 (16.4)	–	53 (26.0)	–	19 (17.9)	35 (27.6)	69 (15.6)
III ~ IV	91 (22.2)	20 (17.2)	–	42 (20.6)	–	17 (16.0)	0	80 (18.1)
NA	0	15 (13.0)		0	–	0	0	28 (6.3)
T stage, *n* (%)								
1	136 (33.3)	–	71 (83.5)	–	–	–	43 (33.9)	–
2	219 (53.7)	–	12 (14.1)	–	–	–	82 (64.6)	–
3	34 (8.3)	–	2 (2.4)	–	–	–	2 (1.6)	–
4	19 (4.7)	–	0	–	–	–	0	–
N stage, *n* (%)								
0	267 (65.4)	–	82 (96.5)	–	–	–	94 (74.0)	–
1 ~ 2	141 (34.6)	–	3 (3.5)	–	–	–	33 (26.0)	–
Overall survival (months)								
Median	32.04 ± 30.68	40.51 ± 35.12	77.84 ± 56.57	58.02 ± 22.53	31.62 ± 19.72	61.17 ± 49.81	48.40 ± 27.23	26.03 ± 13.24
Died (%)	146 (35.8)	66 (57.4)	45 (52.9)	30 (14.7)	32(55.2)	77 (72.6)	51 (40.2)	122 (29.0)

^a^For the GSE3141 dataset, the original data provider did not provide additional demographic or clinical-related data (such as age, stage, etc.), so only the sample size and the available OS data were reported.

### Generation and validation of cholesterol prognostic risk score

After univariate Cox proportional hazards regression model, independent screening and LASSO Cox penalized regression (ISIS-LASSO) and backward stepwise multivariable Cox proportional hazards regression model, only 7 CMRGs significantly associated with LUAD survival were included in the multi-biomarker model, which were used to calculated the cholesterol metabolic-related prognostic risk score (Table S1). According to the coefficient value from polygenic Cox regression analysis, the prognosis risk scoring formulas were calculated as following: Cholescore = 0.286*V (ACOT7) + 0.266*V (ACSL3)−0.173*V (CD79A) + 0.376*V (GALNT2)−0.362*V (TMEM241)−0.230*V (TRIB3) + 0.504*V (UGT2B28). *Cholescore* was cholesterol metabolism-related LUAD prognosis risk score. *V* (*) is the amount of gene expression.

**Fig. 1:**
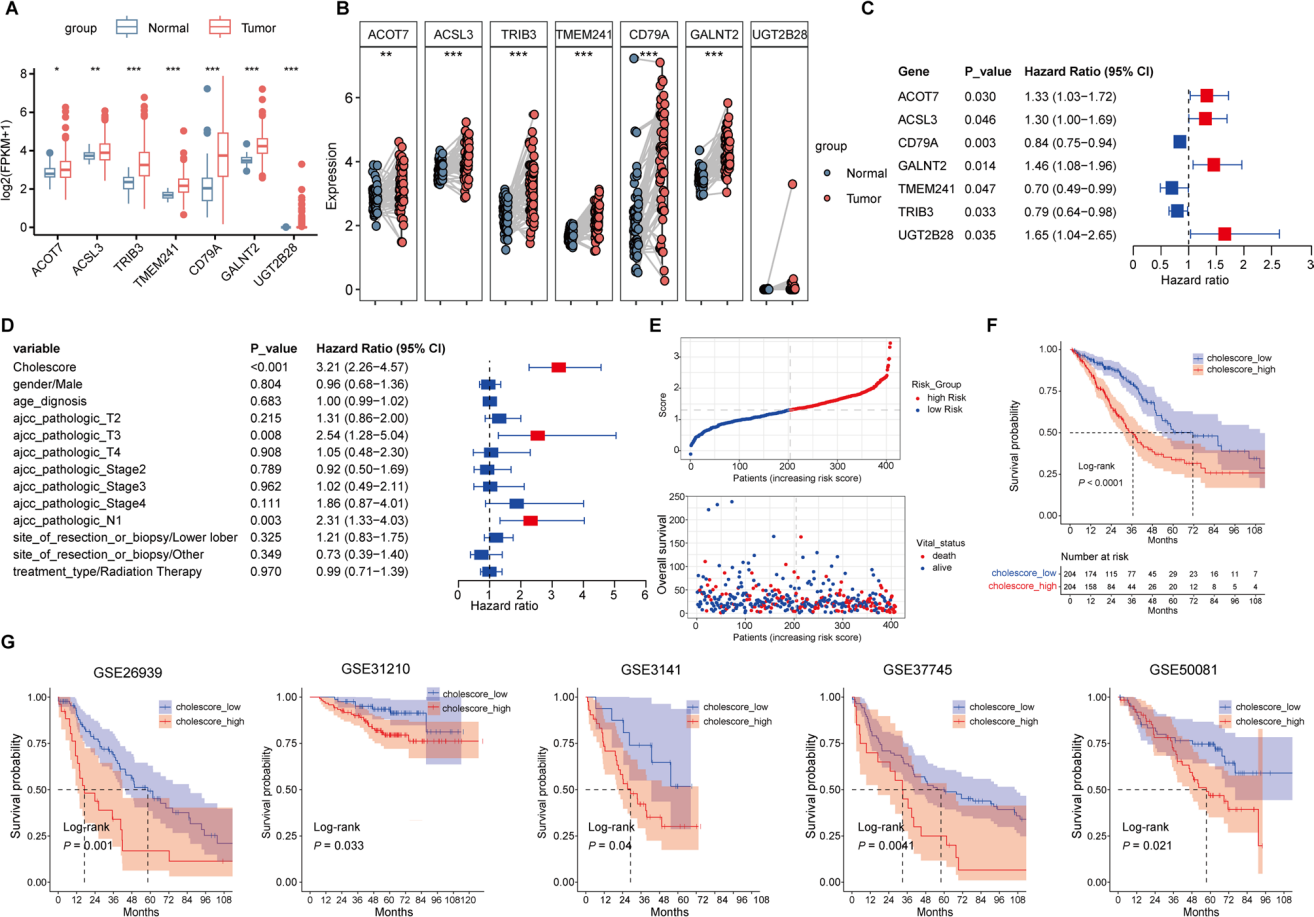
IdentifyingCMRGs associated with LUAD prognosis. (**A** and ** B**) Differential expression of 7 CMRGs between normal and unpaired or paired samples with LUAD patients. (**C**) Forest map of 7 prognostic CMRGs by polygenic Cox analysis. (**D**) Forst map of Cholescore and clinicopathological parameters by multivariate Cox regression analysis. (**E**) Scatter diagram for survival status in Cholescore_low and Cholescore_high TCGA-LUAD patients. (**F**) K-M curve of Cholescore_high and Cholescore_low TCGA-LUAD patients. (**G**) K-M curves of high-Cholescore and low-Cholescore patients in GEO-LUAD cohorts (GSE26939, GSE31210, GSE3141, GSE37745, GSE50081). FPKM = Fragments Per Kilobase of exon model per Million mapped fragments. LUAD = lung adenocarcinoma. *P < 0.05, **P < 0.01, and ***P < 0.001.

The differential expression of 7 CMRGs between normal and unpaired or paired samples with LUAD patients was visualized (Fig. 1A,B). These genes were independently correlative with OS (Fig. 1C). Multivariate Cox proportional hazards regression analyses results showed that the prognostic impact of the Cholescore (Hazard Ratio (HR)_continuous_ = 3.21, 95% Confidence Interval (CI) = [2.26 ~ 4.57], *P* < 0.001; HR_High vs Low_ = 1.93, 95%CI = [1.34 ~ 2.77], *P* < 0.001) remained significant when taking clinical variables into account (Fig. 1D). Patients in training (TCGA) and validation (GEO) sets were categorized into Cholescore_low and Cholescore_high groups based on the median cut-off value of the Cholescore. Obviously, it was observed that more dead patients were distributed in the high Cholescore part in the trianing cohort (Fig. 1E). Survival analysis in the form of Kaplan–Meier curve showed that Cholescore_high patients had significant worse survival than the Cholescore_low patients (Fig. 1F). We then used the same weight coefficients in GSE26939, GSE31210, GSE3141, GSE37745 and GSE50081 as independent validation cohorts and significantly different survival curve difference was also observed (Fig. 1G). Cox proportional regression analysis in the GEO-LUAD cohorts also suggested that Cholescore was an independent prognostic factor for LUAD (Table S2). This indicated that the score had acceptable accuracy in the external validation sets. We further detected whether the Cholescore distributive differences in TCGA-LUAD patients with different clinical characteristics. Higher level of Cholescore preferred to distribute in higher T-stage, N-stage and AJCC stage, and patients with high Cholescores also had a shorter median survival time among stratified patients (Fig. S1).

### Correlation of immune features and Cholescore

**Fig. 2:**
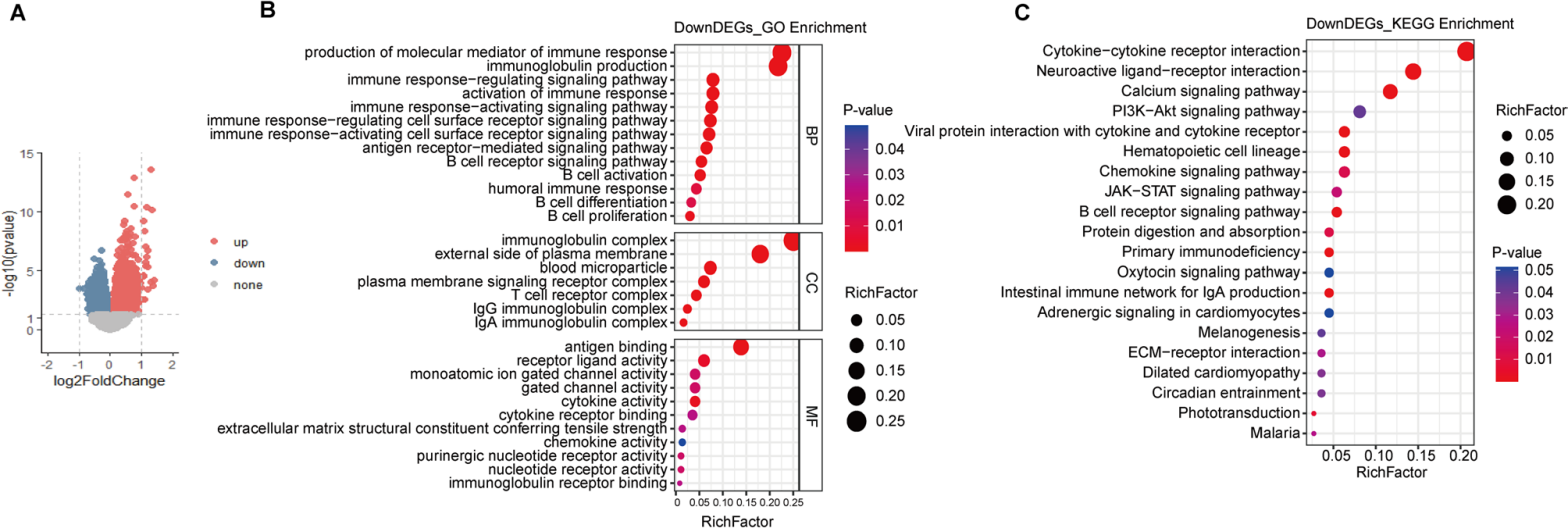
Function analysis of different Cholescore patients of TCGA-LUAD cohort. (**A**) Volcano plots displaying DEGs between Cholescore_low and Cholescore_high patients. (**B** and **C**) Results of GO and KEGG enrichment analyses of DEGs with significantly down-regulated in Cholescore_high patients. DEGs, differentially expressed genes; GO, gene ontology; KEGG, Kyoto encyclopedia of genes and genomes.

The differentially expressed genes (DEGs) between Cholescore_low and Cholescore_high samples were displayed as volcan plot (Fig. 2A). DEGs were identified according to the parameters of a significant difference (*P* < 0.05) and the transformed fold change (Foldchange > 1) of transcripts between two groups. 1789 genes were upregulated, and 1317 genes were downregulated in the Cholescore_high versus Cholescore_low group. These DEGs were selected for enrichment analysis, including Gene Ontology (GO) and Kyoto Encyclopedia of Genes and Genomes (KEGG) enrichment analysis. GO analysis indicated that genes with significantly down-regulated in Cholescore_high patients were mainly involved in immune responses. As for biological processes, they were mainly enriched in several immune-related pathways including “production of molecular medicator of immune response”, “immunoglobulin production”, “immune response-regulating signaling pathway” and “immune response-activating signaling pathway” (Fig. 2B). KEGG analysis also enriched the pathway including “cytokine-cytokine receptor interaction”, “chemokine signaling pathway”, and “B cell receptor signaling pathway” (Fig. 2C).

**Fig. 3:**
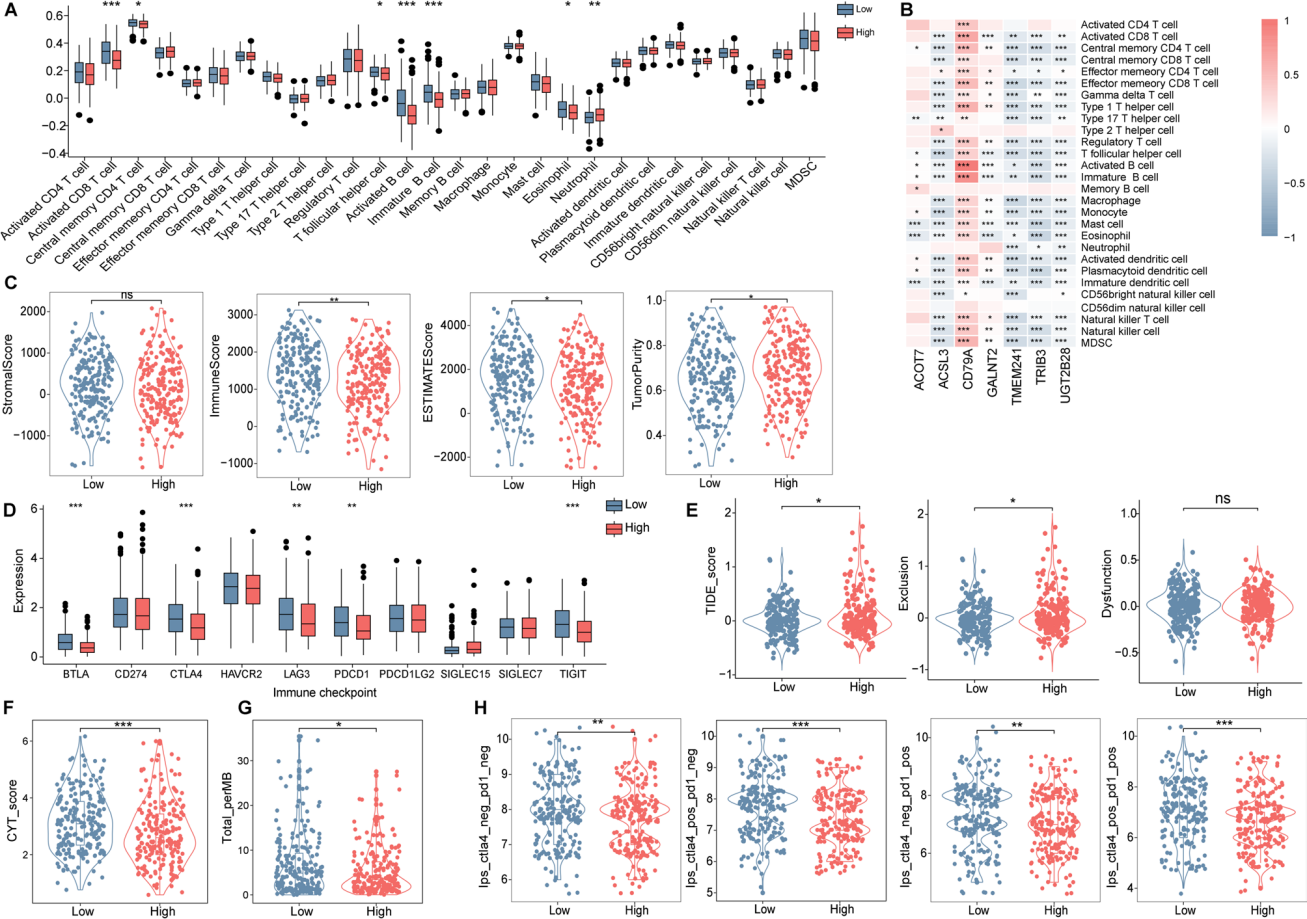
Immune-related analysis in different score subgroups of TCGA-LUAD cohort. (**A**) Comparison of the discrepancy in immune cells infiltration based on ssGSEA. (**B**) Correlation analysis between CMRGs and immune cell infiltration. (**C**) Violin scatter plots comparing stromal score, immune score, estimate score and tumor purity. (**D**) Differential expression of immune checkpoint genes. (**E**) Different distribution of TIDE scores. (**F**) Different distribution of CYT scores. (**G**) Different distribution of TMB scores. (**H**) Different distribution of IPS scores. ssGSEA, single-sample gene set enrichment analysis; TIDE, tumor immune dysfunction and exclusion. ns, not significant, *P < 0.05, **P < 0.01, and ***P < 0.001.

We further analyzed the immune microenvironment in different Cholescore patients. The single-sample gene set enrichment analysis (ssGSEA) was performed for assessing the infiltrating abundance of different immune cells. Subpopulations of adaptive immune lymphocytes involved in recognizing and killing tumor cells including activated CD8 T cell, central memory CD4 T cell, T follicular helper, Activated B cell and immature B cell were less infiltrated in the Cholescore_high patients (Fig. 3A). The model genes were significantly correlated with the proportion of immune cells (Fig. 3B). Infiltrating stromal cells and immune cells constitute the main part of normal cells in tumor tissue. Compared with Cholescore_low patients, the Immune score and Estimate score were lower in the Cholescore_high patients, while the tumor purity was higher (Fig. 3C). This showed that the proportion of immune cells in the tissue samples of the Cholescore_high patients was significantly reduced. We further analyzed the expression of immune checkpoint genes between the two groups, and found that the expression of immune checkpoint genes BTLA, CTLA4, LAG3, PDCD1 and TIGIT were lower in the Cholescore_high patients (Fig. 3D). TIDE score is a tool developed by Peng Jiang^19^ to evaluate the characteristics of tumor immune escape, and a high TIDE score means a high possibility of immune surveillance escape. Results showed that Cholescore_high patients have higher TIDE and exclusion scores (Fig. 3E). CYT score was identified to characterize the anti-tumor immune activity of CD8+ cytotoxic T cells and macrophages^20^. TMB and CYT is significantly associated with significant pan-cancer survival benefits and is effective for anti-CTLA-4 and anti-PD-L1 immunotherapy^21^. Upon examination of the TMB and CYT distribution across patients, we found that the Cholescore_high patients exhibited lower TMB and CYT than patients with Cholescore_low (Fig. 3F,G). Finally, the importance of immunogenicity-based IPS in predicting immunotherapy response in patients with melanoma and colon cancer has been reported^22^. The Ips_ctla4_neg_pd1_neg, Ips_ctla4_pos_pd1_neg, Ips_ctla4_neg_pd1_pos and Ips_ctla4_pos_pd1_pos scores were significantly decreased in Cholescore_high patients (Fig. 3H). This means that the Cholescore_high patients may have a higher possibility of immune surveillance escape and worse success rate of immunotherapy. Finally, we validated in the Memorial Sloan Kettering Cancer Center corhort (GSE248249) and Sjöberg Immune Checkpoint Inhibition cohort (GSE283829) that Cholescore was significantly decreased after PD-L1 treatment; additionally, compared with lung cancer patients with disease progression, those with stable disease also showed a decreasing trend in Cholescore (Fig. S2). Overall, the Cholescore established has great potential in predicting prognosis and immunotherapeutic benefits.

### Generation and validation of Estrogen prognostic risk score

**Fig. 4:**
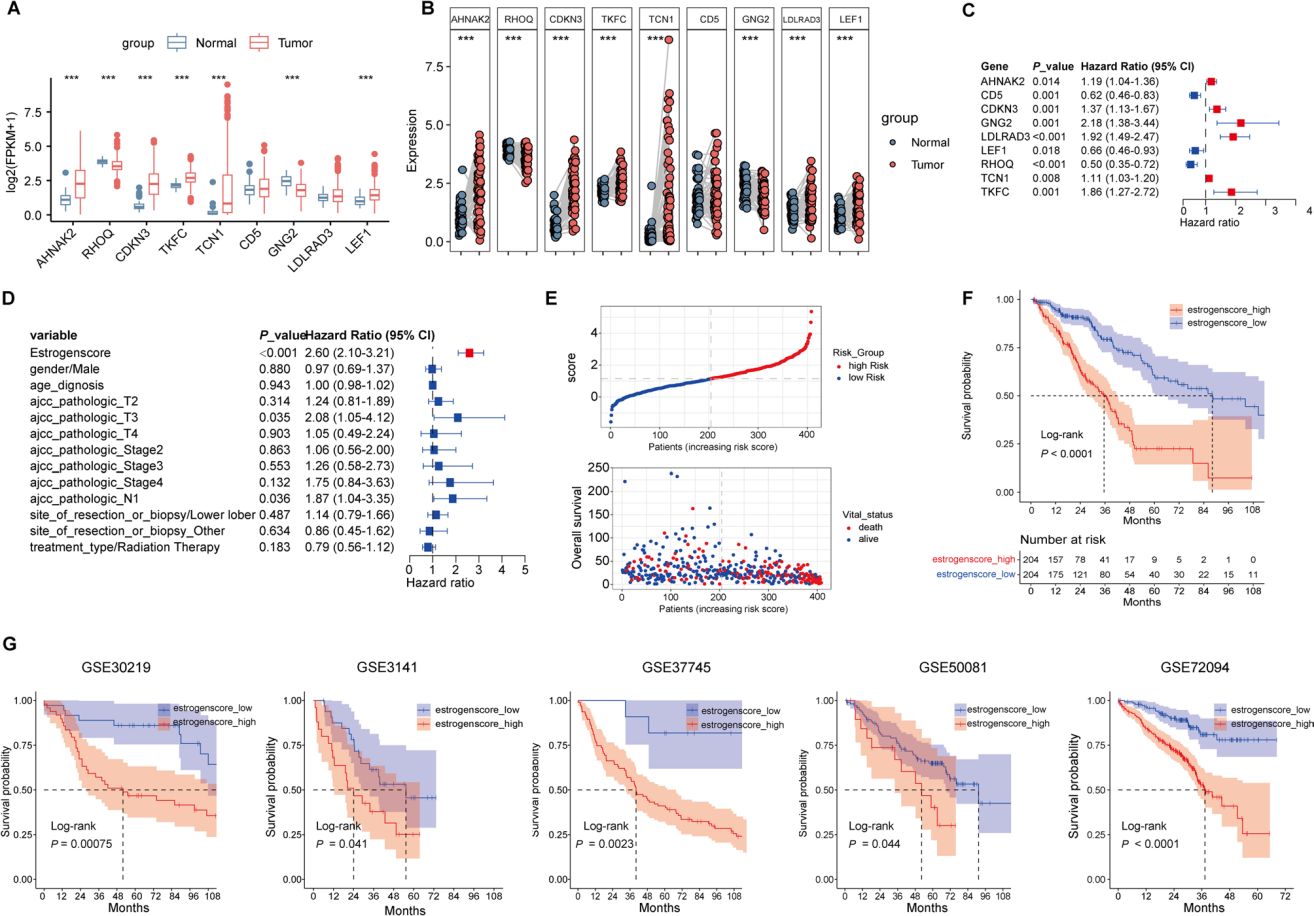
Identifying EMRGs associated with LUAD prognosis. (**A** and **B**) Differential expression of 7 EMRGs between normal and unpaired or paired samples with LUAD patients. (**C**) Forest map of 7 prognostic EMRGs by polygenic Cox analysis. (**D**) Forst map of Estrogenscore and clinicopathological parameters by multivariate Cox regression analysis. (**E**) Scatter diagram for survival status in high-Estrogenscore and low-Estrogenscore TCGA-LUAD patients. (**F**) K-M curve of high-Estrogenscore and low-Estrogenscore TCGA-LUAD patients. (**G**) K-M curves of high-Estrogenscore and low-Estrogenscore patients in GEO-LUAD cohorts (GSE30219, GSE3141, GSE37745, GSE50081, GSE72094). FPKM, fragments per Kilobase of exon model per million mapped fragments, LUAD, lung adenocarcinoma. *P < 0.05, **P < 0.01, and ***P < 0.001.

Our study further revealed that hormone metabolic processes, steroid hormone biosynthesis pathways, estrogen metabolic processes, and steroid metabolic processes are significantly enriched in the high Cholescore group (Table S3). Based on this, we further screened Estrogen metabolism-related genes (EMRGs) associated with LUAD prognosis, and ultimately identified 9 gene probes, which were used as a classifier. Compared with normal tissues, the expressions of AHNAK2, CDKN3, TKFC, TCN1, LDLRAD3 and LEF1 were significantly increased, while the expressions of RHOQ, GNG2 were decreased in LUAD tissues (Fig. 4A,B). Polygenic Cox proportional hazards regression model showed that the regression coefficients of these genes were all statistically significant (Fig. 4C). The scoring formula of Estrogenscore as followed: Estrogenscore = 0.171*V (AHNAK2)−00.478*V (CD5)−0.317*V (CDKN3) + 0.778*V (GNG2) + 0.651*V (LDLRAD3)−0.422*V (LEF1)−0.694*V (RHOQ) + 0.107*V (TCN1) + 0.620*V (TKFC). When the Estrogenscore calculated based on expression values and cox regression coefficients was included in the multivariate Cox proportional hazards regression model including clinical variables, the regression coefficients remained significant (HR_continuous_ = 2.60, 95%CI = [2.10 ~ 3.21], *P* < 0.001; HR_High vs Low_ = 2.85, 95%CI = [1.96 ~ 4.16], *P* < 0.001), proving that the Estrogenscore was an independent prognostic factor for patients with LUAD (Fig. 4D). Survival distributions were plotted in Fig. 4E,F. A better prognosis was observed in the Estrogenscore_low patients both in training and validation cohorts (Fig. 4G). The Cox proportional hazards regression results of EMRGs are detailed in Tables S1 and S2. Similarly, we performed a stratification analysis and found that the risk score groups also present differential survival results in most subgroups (Fig. S3).

### Mediation, interaction and joint analysis of Cholescore and Estrogenscore

We analyzed the association mechanism between the two risk scores and the prognosis of LUAD through causal mediation analysis. The results indicated that approximately 53.99% of the impact of Cholescore on the prognosis of patients is achieved through the indirect effect of Estrogenscore (β for indirect effect = 0.58, 95% CI = [0.37, 0.79], *P* < 0.001; total effect β = 1.07, 95% CI = [0.73, 1.41], *P* < 0.001), and the direct effect of Cholescore on the prognosis of LUAD is also statistically significant (β = 0.51, 95% CI = [0.17, 0.86], *P* = 0.003). This suggests that the Estrogenscore plays an important mediating role in the process of Cholescore influencing the prognosis of patients, and more than half of the total effect is transmitted through this mediating path.

**Fig. 5:**
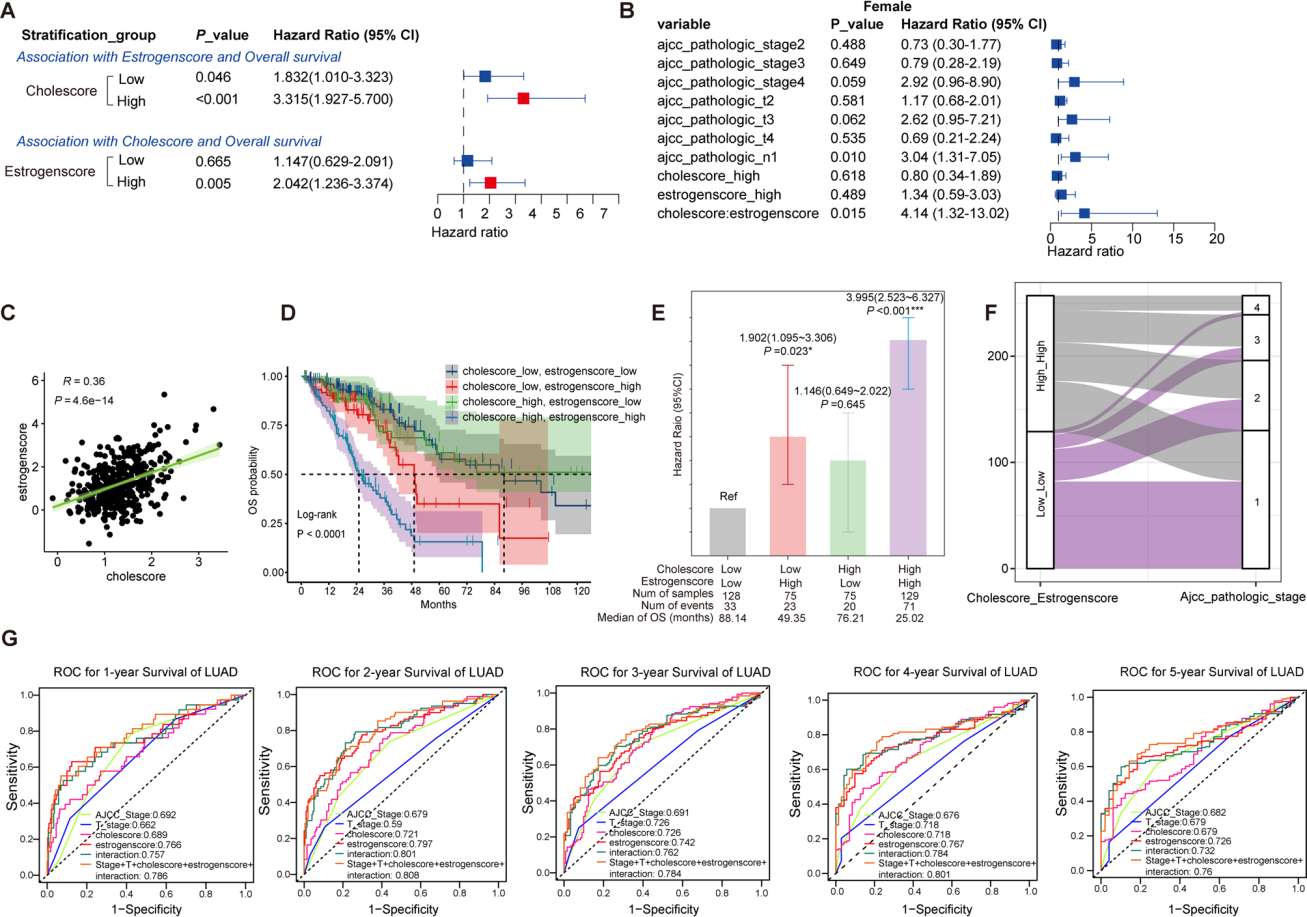
Interaction and joint analysis of Cholescore, Estrogenscore and LUAD prognosis. (**A**) Correlation between risk scores and OS in different score subgroups patients. (**B**) Correlation between clinicopathological features, risk scores and scores-multiplication interactive item and female LUAD prognosis by multivariate Cox regression analysis. (**C**) Correlation between cholesccore and Estrogenscore. (**D**) The K-M curves showing OS in different score subgroups. (**E**) Joint associations of Cholescore and Estrogenscore and OS of TCGA-LUAD patients. (**F**) Sankey plot of stage and different risk scores. (**G**) The 1 ~ 5-year ROC for predicting the sensitivity and specificity of OS according to clinicopathological parameters, Cholescore, Estrogenscore and multiplicative interaction term. *P < 0.05.

The HR (95% CI) between Estrogenscore and LUAD survival was 1.83 (95% CI = [1.01 ~ 3.32]) in the Cholescore_low patients, increased to 3.32 (95% CI = [1.93 ~ 5.70]) in the Cholescore_high individuals (Fig. 5A). Cholescore was significantly associated with LUAD prognosis only in the population with Estrogenscore_high patients (HR = 2.04, 95% CI = [1.24 ~ 3.37], Fig. 5A). Subgroup stratification results were similar for patients with different clinical characteristics (Fig. S4). The results of the interaction effect analysis showed significant multiplicative interaction of Cholescore and Estrogenscore on LUAD prognosis in female and T-stage = 2 patients (Figs. 5B and S5). A positive correlation between Cholescore and Estrogenscore was also detected (Fig. 5C). Subsequently, TCGA-LUAD patients were divided into 4 subgroups according to Cholescore and Estrogenscore. Prognostic analysis showed that choelscore_high and Estrogenscore_high patients had the shortest survival time compared with the other three groups (Fig. 5D). The joint association result showed that compared with Cholescore_low and Estrogenscore_low patients, the HR (95%CI) for those of Cholescore_high and Estrogenscore_high was 4.00 ( [2.53 ~ 6.33], *P* < 0.001, Fig. 5E). Results were not materially changed in sensitivity analysis (Fig. S6). The clinical stage distribution of Cholescore_low and Estrogenscore-low patients tended to be lower stage I (Fig. 5F). In the advanced prediction models for LUAD survival, the combination of the Cholescore, Estrogenscore and the addition of multiplicative interaction terms contributed to the improvement in the prediction accuracy of 1 ~ 5-year OS (Fig. 5G), indicating the usefulness of the selected biomarkers and their interactions in predicting the outcome for patients with LUAD. We also characterized the Gene × Gene interaction effect of CMRGs and EMRGs and found that 6 pairs of gene probes with a Gene × Gene interaction were retained in the multibiomarker model (Table 2).

**Table 2:** Association results of multibiomarker Cox proportional hazards model for 6 significant Gene × Gene interaction terms in training TCGA cohort.

Gene1	Gene2	Multivariable cox		
		β	HR (95% CI)	*P*
ACOT7	RHOQ	0.098	1.103 (1.025 ~ 1.187)	0.008
ACSL3	GNG2	0.147	1.158 (1.040 ~ 1.291)	0.008
CD79A	CD5	− 0.1	0.905 (0.839 ~ 0.975)	0.008
GALNT2	LDLRAD3	0.467	1.595 (1.057 ~ 2.408)	0.026
TMEM241	LDLRAD3	− 0.761	0.467 (0.286 ~ 0.761)	0.002
UGT2B28	TKFC	0.291	1.338 (1.082 ~ 1.655)	0.007

### Threshold effect analysis

We further verified the robustness of the prognostic model through cutoff optimization analyses of the Cholescore and Estrogenscore, including median, tertile, and data-driven optimal cutoffs. First, restricted cubic spline analysis revealed that both the Cholescore and Estrogenscore had a non-linear association with LUAD outcomes, characterized by a continuous increase in risk with higher scores and the presence of a clear threshold effect (Figs. S6A, S6C). Regardless of the grouping strategy used, the high metabolic score group was significantly associated with an increased risk of death from LUAD compared to the low score group (Figs. S6B, S6D). The consistency of survival differences across different cutoffs strongly supports the validity of using the median for risk stratification.

Second, the HRs based on the median (Cholescore: 1.93; Estrogenscore: 2.85) were consistent with those of the optimal cutoffs (Cholescore: 2.94; Estrogenscore: 6.40) in terms of effect direction and statistical significance, with no impact on the core conclusions (Table S4). In terms of predictive performance, the time-dependent area under ROC curve (AUC) values of the median cutoff for the Cholescore remained stable at 0.61–0.67 between 12 and 60 months, only slightly lower than those of the high-risk tertile group (0.65–0.74). For the Estrogenscore, the AUC values based on the median (0.63–0.72) were consistent with those of other cutoffs, all confirming the reliable predictive efficacy of the model (Table S4 and Figs. S6C, S6D). Finally, the median cutoff has prominent clinical utility: as a straightforward and non-arbitrary classification criterion, it can evenly divide the cohort into two groups, making it more convenient for practical application and promotion in clinical settings that prioritize simplicity and universality.

## Discussion

Cholesterol is the major neutral lipid of pulmonary surfactant and is the key to normal lung biology. Lipid-laden macrophage “foam cells” that have been noted in chronic lung disease similarly emphasized the coupling importance of pulmonary cholesterol and immune homeostasis^11,23^. In the lung tumor microenvironment, deficiency of cholesterol efflux pathway in epithelial progenitor cells is the main culprit in the development of early non-small cell lung cancer lesions^12^, and the accumulation of cholesterol in tissues, in addition to providing nutrients to tumor cells, also poses a challenge to the dynamics of the immune microenvironment. However, the role of perturbations of cholesterol balance in the dialogue between immune cells and lung tumor cells is still not well understood. With the in-depth study of the molecular biological mechanisms of lung cancer, the treatment of lung cancer has shown a diversified and precise trend. Targeted therapies for various driver gene mutations^24,25^ such as EGFR, ALK, ROS1, MET, and RET, immunotherapy inhibitors represented by PD-1/PD-L1 antagonists and CTLA-4 antagonists^26^, as well as sensitization by natural plant compounds (such as flavonoids^27^, phytosterols^28^) have continuously made breakthroughs. However, although studies have found that metabolic reprogramming plays an important role in the occurrence and progression of lung cancer, the development and application of drugs targeting metabolic reprogramming in lung cancer treatment have not been satisfactory^29^. Precisely defining the disorder of cholesterol metabolism in lung cancer tissue cells, developing new cholesterol-targeting strategies may improve the effectiveness of lung cancer. In this study, we conducted a two-stage integration study of gene transcriptome expression data from multiple centers to identify novel CMRGs associated with LUAD prognosis. In fact, the risk score we constructed can effectively distinguish survival outcomes of LUAD patients and significantly improve prognosis prediction accuracy. Cholescore’s robustness was also maintained across different cohorts and subgroup people. More importantly, the score was significantly associated with immune signatures and estrogen metabolism.

With the gradual maturity of genetic technology, tumor molecular classification can precisely classify tumors in clinical practice and identify specific key indicators for prognosis evaluation and monitoring by analyzing the characteristics of tumor driver gene variations. Studies have shown that 64% of Chinese cancer patients carry potential clinically treatable genetic mutations^30^, which further highlights the core value of molecular testing in treatment guidance. In the III-stage lung cancer treatment, the multidisciplinary collaboration model enables some inoperable patients to have the opportunity for surgery through neoadjuvant therapy (chemotherapy, targeted or immunotherapy), resulting in a significant reduction in postoperative recurrence rate^31^. This treatment progress is highly correlated with the metabolic score we discovered. The immunological infiltration status in the group with a high metabolic score suggests that they may be more suitable for preoperative combined metabolic regulation therapy to improve the tumor microenvironment. In this context, through liquid biopsy analysis of the gene expression levels in circulating tumor cells or plasma free RNA, based on qPCR technology, the development of detection kits targeting CMRGs and EMRGs has unique clinical translational advantages, including overcoming the limitations of tissue sampling, capturing dynamic molecular characteristics, and supporting precise treatment decisions, etc. This study has screened out key gene targets that are compatible with qPCR detection and have clinical relevance (significantly associated with prognosis and treatment response), which can provide core target references for the development of lung cancer prognosis gene detection kits. Detection systems based on these targets can be combined with existing driver gene mutation detection to directly support the development and transformation of kits, and enhance the ability to analyze the heterogeneity of lung cancer.

Cholesterol consumption is a key feature of tumor-associated macrophages in human LUAD. Our study indicates that in LUAD, the downregulated DEGs of patients with high prognostic risk scores are enriched in pathways related to immune responses. High Cholescore are significantly associated with reduced immune cell infiltration (such as activated CD8⁺ T cells, activated B cells, and eosinophils) and poor response to immune checkpoint inhibitors, highlighting its potential as a marker of the immunosuppressive tumor microenvironment. These findings are consistent with emerging evidence^5,32^ that cholesterol metabolism plays a crucial role in shaping the immune response within the tumor microenvironment, providing mechanistic clues to explain the association between cholesterol scores and immunity observed here. Research evidence suggested that dysregulation of cholesterol metabolism in tumor cells directly impairs the function of immune cells: CD8⁺ T cells are more susceptible to cholesterol deficiency than CD4⁺ T cells^33^. This indicates that the cholesterol metabolism characteristics captured by Cholescore may help cause the exhaustion or dysfunction of T cells in LUAD, explaining the observed reduction in CD8⁺ T cell infiltration in high Cholescore tumors. These findings support the notion that Cholescore likely captures a dual immunosuppressive role of cholesterol metabolism in LUAD: 1) impairing immune cell function through lipid-mediated toxicity, and 2) promoting immune checkpoint activation via oxysterol signaling. Cholescore reflect the cholesterol metabolism status, which actively shapes the immunosuppressive tumor microenvironment in LUAD and ultimately weakens the efficacy of immune checkpoint inhibitor therapy. This highlights the potential for targeting cholesterol metabolism to reverse immune desensitization and improve the immunotherapy outcomes for LUAD patients (those with high cholesterol scores).

Among the seven CMRGs identified in transcriptional analysis, some model genes have been described to be involved in the development of lung cancer and immune. ACOT7 can alter the types of phospholipids^34^, which are the core components of biological membranes. The types, structure and metabolic state of phospholipids profoundly affect cholesterol homeostasis by regulating key processes such as cholesterol synthesis, transport, esterification and excretion. ACOT7 promotes the proliferation of lung cancer cells by inducing the cell cycle through the p53-p21 signaling pathway^35^, regulating cell apoptosis and ferroptosis signals^36^. In mice with knockout of the cholesterol efflux receptors LXRα/β, the expression of ACSL3 is decreased^37^. ACSL3 activates fatty acids to promote β-oxidation, increases the supply of mitochondrial acetyl-CoA, and indirectly enhances the initial step of cholesterol synthesis; at the same time, ACSL3 indirectly affects the generation of phosphatidylcholine by promoting the synthesis of diacylglycerol^38^. PC is a key phospholipid component in the assembly of very low-density lipoprotein (VLDL). Insufficient synthesis of PC can lead to VLDL secretion disorders, thereby affecting the transport of cholesterol from the liver to peripheral tissues. ACSL3 also transports fatty acids to the surface of lipid droplets through the mechanism dependent on Stx17, promoting the synthesis and storage of cholesterol esters^39^. The research has confirmed the crucial role of ACSL3 in driving the progression of LUAD and immune evasion^40^. GALNT2 as a direct modulator of HDL metabolism across mammals. GALNT2 knockdown repressed NSCLC cell proliferation, migration, and invasion and restrained tumor formation in nude mice^41,42^. A new study has found that TMEM241 involves in M6P modification of the cholesterol lysosomal transporter NPC2, and its loss leads to the accumulation of lysosomal cholesterol in the lung, which is characterized by lung damage and decreased motor function^43^. TRIB3 has also been shown to be associated with macrophage polarization^44^, and UGT2B28 is a regulatory factor in the steroid synthesis process^45^. However, there have been no studies involving the regulatory role of these model genes in lung cancer immune. These novel cholesterol regulatory genes may provide new perspectives for future lung cancer cholesterol and immune research.

Currently, the lung cancer prognosis risk stratification models constructed at the molecular level have comprehensively covered the core biological characteristics of the tumor, including the abnormal regulation of metabolic-related genes^46^, the expression patterns of epigenetic markers such as methylation^47,48^, and the cell death-related markers^49,50^, providing an important tool for analyzing the heterogeneity of lung cancer. However, it is worth noting that most of the existing models focus on a single molecular pathway or feature, and pay insufficient attention to the synergistic regulatory effects of different pathways in the metabolic network, especially cholesterol and estrogen. The intrinsic association between them in biosynthesis and signal transduction has not been fully integrated into the lung cancer prognosis assessment. These further prompts us to consider developing an interaction model of two metabolic scores for lung cancer prognosis risk stratification, to capture the synergistic effect of the “cholesterol—estrogen” metabolic network on the prognosis of lung cancer, and improve the biological rationality and clinical accuracy of risk stratification. Our research shows that in the high Cholescore group, the estrogen metabolism process, steroid metabolism process, and steroid hormone biosynthesis pathway are significantly enriched, and the mediation analysis and interaction analysis also fully confirm the cross-talk effect between the two scores. This result may reveal that abnormal cholesterol metabolism may form a cascade effect by driving the activation of the estrogen-related metabolic network, and further suggests that the synergistic regulation of the two may be an important potential mechanism affecting the prognosis of lung cancer. In fact, some existing studies have confirmed that the combination of metabolic characteristics of cholesterol and estrogen exerts synergistic effects in EGFR-TKI resistance in lung cancer^51^ and prognosis of breast cancer^52^. These studies provide a solid theoretical basis for us to develop the interactive model of the two metabolic scores, and further support the value of integrating multiple metabolic pathways in clinical decision-making for LUAD. The result of Gene × Gene interactions might also provide clues for further research. Taking the gene pair ACSL3-GNG2 as an example, as mentioned above, ACSL3 was involved in the regulation of cholesterol metabolism through multiple mechanisms, with its roles spanning key processes such as cholesterol synthesis, transport, and efflux. Meanwhile, studies have found that ACSL3 silencing can inhibit estrogen secretion in goose hierarchical follicular granulosa cells^53^ and suppress the growth of breast cancer cell line MCF-7^54^. As a component of the Gβγ subunit, GNG2 participates in the signal transduction of G protein-coupled receptors. The interaction between GNG2 and the muscle RAS oncogene homolog may inhibit the activity of Akt and ERK, thereby suppressing the proliferation of estrogen-dependent breast cancer cells^55^. The Akt pathway can upregulate the transcription and translation of ACSL3^56^. For ACOT7-RHOQ, Inhibiting the expression of RHOQ can promote TGF-β-mediated EMT and the invasion of LUAD cells^57^. RHOQ is a Rho family GTPase with a primary sequence and participates in insulin-stimulated glucose uptake in adipocytes^58^. Insulin resistance in adipocytes leads to an increase in paracrine estrogen release^59^, and ACOT7 is necessary for normal glucose tolerance and insulin secretion^60^. Thus, it is speculated that these genes may jointly influence the progression of lung cancer through signal crossover, cholesterol and estrogen metabolism synergy, etc. The synergistic effect between genes suggests that they may become combined treatment targets, but further research is needed to clarify the details of the interaction and the tissue-specific mechanisms.

The unique value of this study lies in the systematic exploration of the impact of cholesterol metabolism on the prognosis of LUAD, along with in-depth analysis of its mechanism of action in the tumor immune microenvironment and its crosstalk regulatory relationships with estrogen metabolism. This provides a new perspective for understanding the metabolic-immune regulatory network in LUAD. Meanwhile, we are clearly aware of the limitations of the current study: First, the data were derived from public databases, and the sample size of the validation cohorts needs to be expanded. Second, although the association between Cholescore and treatment response has been preliminarily validated in two independent immunotherapy cohorts (GSE248249 and GSE283829), prognostic validation based on large scale clinical surgical samples is still needed. With the rapid development of cancer omics detection technologies and biochip technologies, we believe that by integrating multi-dimensional molecular data with clinical information, the model constructed in this study will gradually overcome existing technical limitations, ultimately achieving translation into clinical practice and providing a practical tool for prognostic assessment and guidance for immunotherapy in LUAD.

## Conclusions

The cholesterol metabolic risk score (Cholescore) constructed has great potential in predicting LUAD prognosis and immunotherapeutic benefits, and combination of cholesterol and estrogen risk scores might significantly improve the accuracy of survival prediction in patients with LUAD.

## Material and methods

### Patients and collection

We collected the RNA-seq data and clinical information from TCGA and GEO databases respectively. Inclusion and exclusion criteria: patients without survival time, follow up < 30 days, prior malignancy = ”yes” and replicated sequenced were excluded, a total of 408 TCGA-LUAD patients with full RNA expression, survival time (OS) and covariates data were included for the screening of prognosis biomarkers in the discovery phase. Transcriptome information with LUAD cases was profiled using the Affymetrix Human Genome U133A plus 2.0 Array (GSE32019, GSE31210, GSE3141, GSE37745, GSE50081), the Agilent-UNC-custom-4X44K (GSE26939) and the Rosetta/Merck Human RSTA Custom Affymetrix 2.0 microarray (GSE72094) from GEO database for model validation.

### Development of metabolism-related prognostic risk scores

We used the following keywords “cholesterol” and “cholesterol metabolism” for exposure and collected CMRGs from the Molecular Signature Database (MSigDB). After removing the overlapped gene, 586 CMRGs were obtained. The end OS was determined by time of death. The transcription profile of CMRGs were used as prognosis predictor. CMRGs associated with LUAD survival were screened by using univariate, ISIS-LASSO and multivariate Cox regression analyses in turn. ISIS-LASSO filters variables based on Akaike information criterion. Multivariate Cox proportional hazards model applied a backward stepwise regression strategy was used to estimate the hazard ratios (HRs) and 95% confidence intervals of CMRGs associated with OS. The P value thresholds for multiple testing were established by the Bonferroni method with the overall type I error would be controlled at the 0.05 level. Prognostic risk scores (Cholescore) were calculated on the basis of a weighted linear combination of individual values of the gene expression, with weights derived from multivariate Cox proportional hazards regression model. The process of estrogen metabolism-related genes (EMRGs) screening and risk score (Estrogenscore) construction related to LUAD prognosis was the same as above.

### Enrichment analysis and immune analysis

TCGA samples were divided into two groups based on Cholescore median, and the differentially expressed genes (DEGs) were analyzed. GO and KEGG enrichment analysis of DEGs were performed.

ssGSEA algorithm was employed to quantify the relative abundance of each type of immune cell infiltration within the tumor microenvironment (TME). The gene sets used to characterize distinct immune cell infiltration phenotypes in the TME were sourced from a study published by Charoentong et al. in cell reports^61^. This work provides gene sets corresponding to a broad spectrum of human immune cell subtypes, including activated CD8+ T cells, activated dendritic cells, macrophages, natural killer T cells, and regulatory T cells, among others. Spearman correlation analysis was used to analyze the correlation between CMRGs and immune cells. Immune score, stromal score, estimate score and tumor purity to evaluate the immune infiltration^62^. The differences in immune checkpoints among different Cholescore patients were analyzed to predict sensitivity of immunotherapy. Immune-related features, including immune cytolytic activity (CYT), tumor mutation burden (TMB) and immunogenicity-based Immunophenoscores (IPS) were also retrieved. The CYT score was calculated using the geometric mean of GZMA and PRF1 expression. TMB was based on whole-exome sequencing data from the TCGA-LUAD cohort. The variant filtering criteria were as follows: synonymous mutations, germline mutations, and mutations with an allele frequency < 0.05 were excluded, while missense mutations, nonsense mutations, frameshift mutations, and other gain-of-function or loss-of-function mutations were retained. Immunophenoscores (IPS) of TCGA-LUAD patients were obtained from the Cancer Immunome Atlas (TCIA) database^63,64^. Tumor immune dysfunction and rejection (TIDE) score predicted the possibility of tumor immune escape and the effect of immune checkpoint inhibition.

### Interaction analysis

Mediating effect of Cholescore and Estrogenscore was evaluated on the LUAD prognosis were evaluated in TCGA base. Cox proportional hazards regression model by creating a product term to analyze multiplicative interactions. Prognostic HRs for LUAD were calculated according to the four groups of population with different Cholescore (low, high) and Estrogenscore (low, high) to evaluate the joint association. The advanced models were constructed by combining risk scores and clinical features associated with prognosis. The time-dependent ROC was used to describe the specificity and sensitivity of the prediction model (risk score prognosis model, clinical features prognosis model and risk scores combined with clinical features prognosis models), the area under ROC curve (AUC) was used to measure the accuracy of the prediction model. Multivariate Cox proportional hazards model adjusted for covariates was applied to identify biomarkers with Gene × Gene interactions.

### Statistical analyses

Patient in training (TCGA) and validation (GEO) cohorts were divided into two groups based on the median value of Cholescore. Kaplan–Meier curves were drawn to compare the survival difference of patients with different risk scores. Stratified sensitivity analysis was performed by gender, age at diagnosis, clinical stage, T stage, N stage and AJCC clinical stage to compare the survival differences of different risk scores among different subgroups. A restricted cubic spline model was used to analyze the non-linear association between metabolic scores and the prognosis of LUAD, and the robustness of the prognostic model was validated through cutoff optimization analyses, including median, tertile, and data-driven optimal cutoffs. Among these, the data-driven optimal cutoff is defined as the critical value that maximizes the difference in survival outcomes between different groups of samples.

Batch effect correction was performed on the transcriptomic data of TCGA and GEO cohort using the ComBat() function from the *sva* package. *ISIS-LASSO* analysis was conducted using *SIS* package*.* Kaplan–Meier curves, log rank *P* values, and Cox-proportional hazard ratios were generated using the *survival* and *survminer* packages. DEGs were obtained using *Deseq2* package. “*clusterProfiler*” packages was used to do GO and KEGG enrichment analysis. “*GSVA*” and “*estimate*” packages were used to do ssGSEA and ESTIMATE analysis. “*Maftools*” package was used to analyze genomic mutation data. The mediating effect were evaluated by “*mets*” package. Time-dependent AUC of the ROC were calculated using “*survivalROC*” and “*timeROC*” packages. Spearman’s rank correlation coefficient was calculated using “*cor*” and “*cor.test*” packages. All analyses were performed using R version 4.3.0. All the mentioned packages and functions are included in the R programming language. We considered two-sided *P* values < 0.05 to be significant.

## Supplementary Information

Below is the link to the electronic supplementary material.

Supplementary Material 1

## Data Availability

Data derived from public domain resources. The data that support the findings of this study are available in the TCGA repository (https://www.cancer.gov/ccg/research/genome-sequencing/tcga), GSE26939 (https://www.ncbi.nlm.nih.gov/geo/query/acc.cgi?acc=GSE26939), GSE30219 (https://www.ncbi.nlm.nih.gov/geo/query/acc.cgi?acc=GSE30219), GSE31210 (https://www.ncbi.nlm.nih.gov/geo/query/acc.cgi?acc=GSE31210), GSE3141 (https://www.ncbi.nlm.nih.gov/geo/query/acc.cgi?acc=GSE3141), GSE37745 (https://www.ncbi.nlm.nih.gov/geo/query/acc.cgi?acc=GSE37745), GSE50081 (https://www.ncbi.nlm.nih.gov/geo/query/acc.cgi?acc=GSE50081), GSE72094 (https://www.ncbi.nlm.nih.gov/geo/query/acc.cgi?acc=GSE72094), GSE248249 (https://www.ncbi.nlm.nih.gov/geo/query/acc.cgi?acc=GSE248249), GSE283829(https://www.ncbi.nlm.nih.gov/geo/query/acc.cgi?acc=GSE283829), MsigDB dataset (https://www.gsea-msigdb.org/gsea/msigdb/index.jsp), TCIA, (https://tcia.at/home). The gene sets used in ssGSEA to characterize each type of immune cell infiltrating the TME were derived from the study published by Charoentong, P., et al. in Cell Reports in 2017 (10.1016/j.celrep.2016.12.019). The detailed information of these gene sets is clearly specified in Supplementary Table S6 of the original publication.
