# Supplementary material for: Independent validation of lung adenocarcinoma prognostic risk scores incorporating cholesterol and estrogen metabolism related transcriptional biomarkers
Source: Sci Rep. 2025 Oct 31;15:38159. doi: 10.1038/s41598-025-22140-w (PMC12578827; doi:10.1038/s41598-025-22140-w)

***Supplementary materials***

**Independent Validation of Lung Adenocarcinoma Prognostic Risk Scores Incorporating Cholesterol and Estrogen Metabolism Related Transcriptional Biomarkers**

Qian Zhu^1,2,3^, Yuemei Zhang^1,2^, Jian Ma^2^, Yongjia Li^2^, Hongya Liu^2^, Zhongwen Gong^2^, Ming Du^4*^, Xuemei Lian^1,2*^

^1^ Center for Lipid Research, Key Laboratory of Molecular Biology for Infectious Diseases (Ministry of Education), Chongqing Medical University

^2^ Department of Nutrition and Food Hygiene, College of Public Health, Chongqing Medical University, Chongqing 400016, China

^3^Clinical Medical Research Center, Meteorological Medical Research Center, Panzhihua Central Hospital, 617000 Sichuan, China

^4^Department of Cardiothoracic Surgery, The Second Affiliated Hospital, Chongqing Medical University, Chongqing 400010, China

**Table S1** Association of CMRGs and EMRGs with prognosis of lung adenocarcinoma in training TCGA-LUAD patients.

| Gene | Description | Univariate Cox | | |  | Multivariable Cox† | | |
| --- | --- | --- | --- | --- | --- | --- | --- | --- |
| **Cholesterol metabolism-related gene probes** | | β | HR (95% *CI*) | *P* |  | β | HR (95% *CI*) | *P* |
| ACOT7 | Acyl-CoA thioesterase 7 | 0.378 | 1.46 (1.05~2.03) | 0.024 |  | 0.286 | 1.33 (1.03~1.72) | 0.030 |
| ASCL3 | Achaete-scute family bHLH transcription factor 3 | 0.378 | 1.46 (1.05~2.02) | 0.023 |  | 0.266 | 1.30 (1.004~1.69) | 0.046 |
| CD79A | CD79a molecule | -0.338 | 0.71 (0.51~0.99) | 0.043 |  | -0.173 | 0.84 (0.75~0.94) | 0.003 |
| GALNT2 | Polypeptide N-acetylgalactosaminyltransferase 2 | 0.386 | 1.47 (1.06~2.04) | 0.020 |  | 0.376 | 1.46 (1.08~1.96) | 0.014 |
| TMEM241 | Transmembrane protein 241 | -0.409 | 0.66 (0.48~0.92) | 0.014 |  | -0.362 | 0.70 (0.48~0.99) | 0.047 |
| TRIB3 | Ttribbles pseudokinase 3 | 0.342 | 1.41 (1.01~1.95) | 0.041 |  | -0.230 | 0.79 (0.64~0.98) | 0.033 |
| UGT2B28 | UDP glucuronosyltransferase family 2 member B28 | -0.364 | 0.69 (0.49~0.98) | 0.039 |  | 0.504 | 1.65 (1.03~2.64) | 0.035 |
|  |  |  |  |  |  |  |  |  |
| **Estrogen metabolism -related gene probes** | | β | HR (95% *CI*) | *P* |  | β | HR (95% *CI*) | *P* |
| AHNAK2 | AHNAK nucleoprotein 2 | 0.417 | 1.52 (1.09~2.10) | 0.012 |  | 0.171 | 1.19 (1.03~1.36) | 0.014 |
| CD5 | CD5 molecule | -0.477 | 0.62 (0.45~0.86) | 0.004 |  | -0.478 | 0.62 (0.46~0.83) | 0.001 |
| CDKN3 | Cyclin dependent kinase inhibitor 3 | 0.536 | 1.71 (1.22~2.39) | 0.002 |  | 0.317 | 1.37 (1.13~1.67) | 0.001 |
| GNG2 | G protein subunit gamma 2 | -0.360 | 0.70 (0.50~0.97) | 0.033 |  | 0.778 | 2.18 (1.38~3.44) | <0.001 |
| LDLRAD3 | Low density lipoprotein receptor class A domain containing 3 | 0.613 | 1.85 (1.33~2.57) | <0.001 |  | 0.651 | 1.92 (1.49~2.47) | <0.001 |
| LEF1 | Lymphoid enhancer binding factor 1 | -0.339 | 0.71 (0.51~0.99) | 0.041 |  | -0.422 | 0.66 (0.46~0.93) | 0.018 |
| RHOQ | Ras homolog family member Q | -0.415 | 0.66 (0.48~0.91) | 0.013 |  | -0.694 | 0.50 (0.35~0.72) | <0.001 |
| TCN1 | Transcobalamin 1 | 0.399 | 1.49 (1.08~2.06) | 0.016 |  | 0.107 | 1.11 (1.03~1.20) | 0.007 |
| TKFC | Triokinase and FMN cyclase | 0.411 | 1.51 (1.09~2.09) | 0.014 |  | 0.620 | 1.86 (1.27~2.72) | 0.001 |

†: Cholesterol prognosis model adjusted genes: ACAT1, ACOT7, ACSL3, B4GALT1, BTK, CD79A, CH25H, CPEB2, CYP17A1, EMD, FABP5, FOSL1, GALNT2, GPLD1, IL17F, KIF12, LCK, MAOB, MYLIP, NUP107, PLA2G, RXRB, TMEM241, TRIB3, UGT2B28, UGT2B4; Estrogen prognosis model adjusted genes: AHNAK2, ASAP3, CD5, CDKN3, CYP17A1, GNG2, GRIK3, IL16, KAT2B, LDLRAD3, LEF1, PXK, RHOQ, RTN1, TCN1, TKFC.

**Table S2** Association results of Cholescore and Estrogenscore in univariate and multivariable Cox proportional hazards regression model in validation phase from GEO databases.

| Datasets | Univariate Cox | |  | Multivariable Cox† | |
| --- | --- | --- | --- | --- | --- |
| **Cholesterol prognosis model validation datasets** | | |  |  | |
|  | HR (95% *CI*) | *P* |  | HR (95% *CI*) | *P* |
| GSE26939 | 2.38 (1.40~4.04) | 0.001 |  | 2.56 (1.34~4.87) | 0.004 |
| GSE31210 | 2.44 (1.05~5.68) | 0.039 |  | 1.82 (0.79~4.63) | 0.15 |
| GSE3141 | 2.48 (1.01~6.06) | 0.047 |  | - | - |
| GSE37745 | 2.16 (1.26~3.70) | 0.005 |  | 2.28 (1.31~3.95) | 0.003 |
| GSE50081 | 1.94 (1.10~3.43) | 0.023 |  | 1.85 (0.99~3.42) | 0.0518 |
| **Estrogen prognosis model validation datasets** | | |  |  | |
|  | HR (95% *CI*) | *P* |  | HR (95% *CI*) | *P* |
| GSE30219 | 3.01 (1.54~5.90) | 0.001 |  | 2.82 (1.40~5.68) | 0.004 |
| GSE3141 | 2.04(1.02~4.10) | 0.045 |  | - | - |
| GSE37745 | 6.64(1.63~27.06) | 0.008 |  | 6.15 (1.50~25.23) | 0.012 |
| GSE50081 | 1.97(1.00~3.87) | 0.048 |  | 2.37 (1.11~5.04) | 0.025 |
| GSE70294 | 3.46 (2.12~5.63) | <0.001 |  | 2.83 (1.65~4.86) | <0.001 |

†: adjusted variable: GSE26939: age, gender, bronchioloalveolar content, grade; GSE30219: age at surgery, gender, T stage, N stage; GSE31210: age, gender, smoking status, stage; GSE3141: no adjustment due to the dataset didn’t provide information on clinical variables; GSE37745: age, gender, stage; GSE50081: age, gender, smoking, stage, T stage, N stage; GSE70294: gender, smoking status, stage.

**Table S3** Enriched signaling pathways of upregulated DEGs in high-Cholescore patients.

| ID | Description | P-value | P-adjust | Count |
| --- | --- | --- | --- | --- |
| **TOP 20 KEGG Enrichment** | |  |  |  |
| hsa00830 | Retinol metabolism | 7.72E-15 | 1.90E-12 | 21 |
| hsa00982 | Drug metabolism - cytochrome P450 | 2.79E-14 | 3.44E-12 | 21 |
| hsa04976 | Bile secretion | 2.89E-13 | 2.37E-11 | 22 |
| hsa00980 | Metabolism of xenobiotics by cytochrome P450 | 1.71E-12 | 1.05E-10 | 20 |
| hsa00140 | Steroid hormone biosynthesis | 2.24E-12 | 1.10E-10 | 18 |
| hsa00040 | Pentose and glucuronate interconversions | 4.54E-12 | 1.86E-10 | 14 |
| hsa00053 | Ascorbate and aldarate metabolism | 7.83E-12 | 2.75E-10 | 13 |
| hsa05204 | Chemical carcinogenesis - DNA adducts | 1.67E-11 | 5.13E-10 | 18 |
| hsa00860 | Porphyrin metabolism | 1.19E-10 | 3.24E-09 | 14 |
| hsa04080 | Neuroactive ligand-receptor interaction | 2.37E-08 | 5.84E-07 | 36 |
| hsa05207 | Chemical carcinogenesis - receptor activation | 4.51E-07 | 1.01E-05 | 24 |
| hsa00983 | Drug metabolism - other enzymes | 6.95E-07 | 1.42E-05 | 14 |
| hsa00512 | Mucin type O-glycan biosynthesis | 2.96E-05 | 0.000559 | 8 |
| hsa05150 | Staphylococcus aureus infection | 3.19E-05 | 0.00056 | 13 |
| hsa01240 | Biosynthesis of cofactors | 0.000103 | 0.001682 | 16 |
| hsa02010 | ABC transporters | 0.000159 | 0.00245 | 8 |
| hsa04950 | Maturity onset diabetes of the young | 0.000243 | 0.003511 | 6 |
| hsa04979 | Cholesterol metabolism | 0.000391 | 0.005347 | 8 |
| hsa04061 | Viral protein interaction with cytokine and cytokine receptor | 0.000809 | 0.010475 | 11 |
| hsa04610 | Complement and coagulation cascades | 0.0009 | 0.011071 | 10 |
| **TOP 20 GO Enrichment: biological process pathways** | | | |  |
|  | Description | P-value | P-adjust | Count |
| GO:0006805 | xenobiotic metabolic process | 3.25E-15 | 1.27E-11 | 27 |
| GO:0052695 | cellular glucuronidation | 7.71E-15 | 1.34E-11 | 13 |
| GO:0007586 | digestion | 1.03E-14 | 1.34E-11 | 28 |
| GO:0042445 | hormone metabolic process | 1.57E-13 | 1.53E-10 | 35 |
| GO:0006063 | uronic acid metabolic process | 3.39E-13 | 2.20E-10 | 13 |
| GO:0019585 | glucuronate metabolic process | 3.39E-13 | 2.20E-10 | 13 |
| GO:0019730 | antimicrobial humoral response | 4.01E-13 | 2.23E-10 | 25 |
| GO:0071466 | cellular response to xenobiotic stimulus | 8.44E-13 | 4.11E-10 | 30 |
| GO:0008544 | epidermis development | 4.47E-12 | 1.91E-09 | 42 |
| GO:0022600 | digestive system process | 4.90E-12 | 1.91E-09 | 22 |
| GO:0061844 | antimicrobial humoral immune response mediated by antimicrobial peptide | 1.52E-11 | 5.32E-09 | 19 |
| GO:0008210 | estrogen metabolic process | 1.64E-11 | 5.32E-09 | 14 |
| GO:0008202 | steroid metabolic process | 1.80E-10 | 5.40E-08 | 36 |
| GO:0030277 | maintenance of gastrointestinal epithelium | 6.41E-10 | 1.79E-07 | 10 |
| GO:0043588 | skin development | 7.51E-10 | 1.95E-07 | 34 |
| GO:0031424 | keratinization | 1.40E-09 | 3.41E-07 | 17 |
| GO:0009913 | epidermal cell differentiation | 7.11E-09 | 1.61E-06 | 28 |
| GO:0009410 | response to xenobiotic stimulus | 7.42E-09 | 1.61E-06 | 40 |
| GO:0045109 | intermediate filament organization | 1.24E-08 | 2.55E-06 | 15 |
| GO:0006959 | humoral immune response | 1.75E-08 | 3.41E-06 | 28 |

**Table S4** the results of the truncated optimization analysis of cholesterol scores and estrogen scores (including the median, quartiles, and the optimal truncation values based on the data).

| **Cutoff** | Cutoff | HR (95%CI) | P value | AUC | | | | |
| --- | --- | --- | --- | --- | --- | --- | --- | --- |
|  |  |  |  | 12 | 24 | 36 | 48 | 60 |
| **Median** |  |  |  |  |  |  |  |  |
| (High vs low) | 1.31 | 1.93(1.34~2.77) | <0.001 | 0.61 | 0.67 | 0.66 | 0.65 | 0.60 |
| **Tertile** |  |  |  |  |  |  |  |  |
| (Medium vs low) | 1.08 | 1.42(0.88~2.29) | 0.15 | 0.54 | 0.61 | 0.66 | 0.61 | 0.56 |
| (High vs low) | 1.52 | 2.85 (1.80~4.51) | <0.001 | 0.65 | 0.70 | 0.72 | 0.74 | 0.70 |
| **Optimal** |  |  |  |  |  |  |  |  |
| (High vs low) | 1.59 | 2.94 (2.03~4.24) | <0.001 | 0.64 | 0.65 | 0.65 | 0.67 | 0.66 |
|  | | | | | | | | |
| **Estrogenscore** |  |  |  |  |  |  |  |  |
| **Median** |  |  |  |  |  |  |  |  |
| (High vs low) | 1.15 | 2.85(1.96~4.16) | <0.001 | 0.63 | 0.70 | 0.67 | 0.72 | 0.71 |
| **Tertile** |  |  |  |  |  |  |  |  |
| (Medium vs low) | 0.75 | 1.11(0.68~1.82) | 0.67 | 0.47 | 0.57 | 0.60 | 0.53 | 0.50 |
| (High vs low) | 1.55 | 3.77(2.41~5.90) | <0.001 | 0.69 | 0.74 | 0.77 | 0.72 | 0.75 |
| **Optimal** |  |  |  |  |  |  |  |  |
| (High vs low) | 2.06 | 6.40(4.26~9.60) | <0.001 | 0.75 | 0.73 | 0.68 | 0.69 | 0.66 |

**Figure S1** Survival analysis of Cholescore and LUAD overall survival in different subgroup patients. Differential expression scatter plot and K-M curves of Cholescore and LUAD overall survival in different gender (A), age at diagnosis (B), T stage (C), N stage (D) and pathologic stage (E) patients. NS = no significant, *p< 0.05, **p< 0.01, and ***p< 0.001.


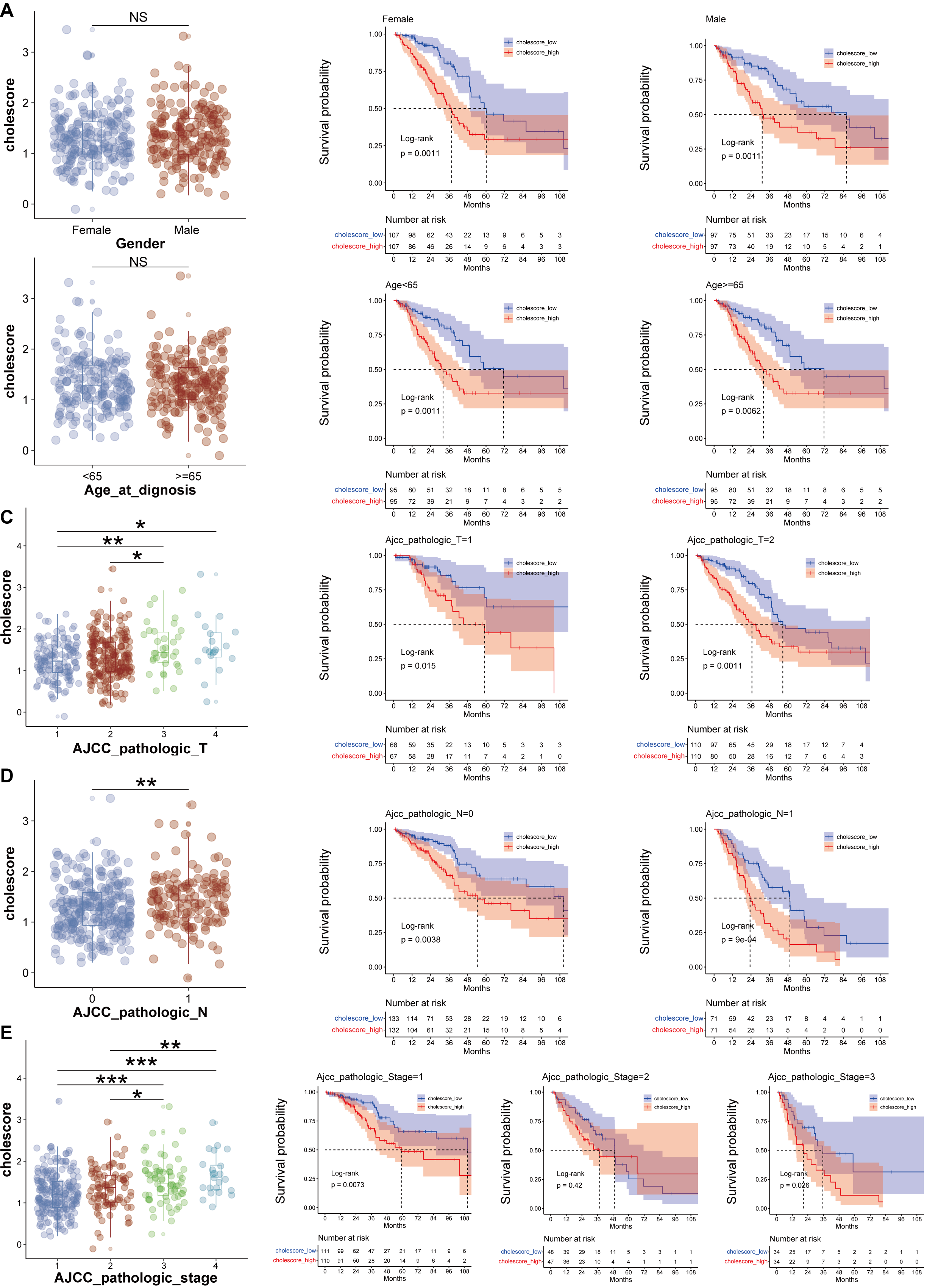


**Figure S2** The validation of Cholescore in the immunotherapy cohort.(A)gse248249 and (B)GSE283829. *p< 0.05, **p< 0.01, and ***p< 0.001.

**
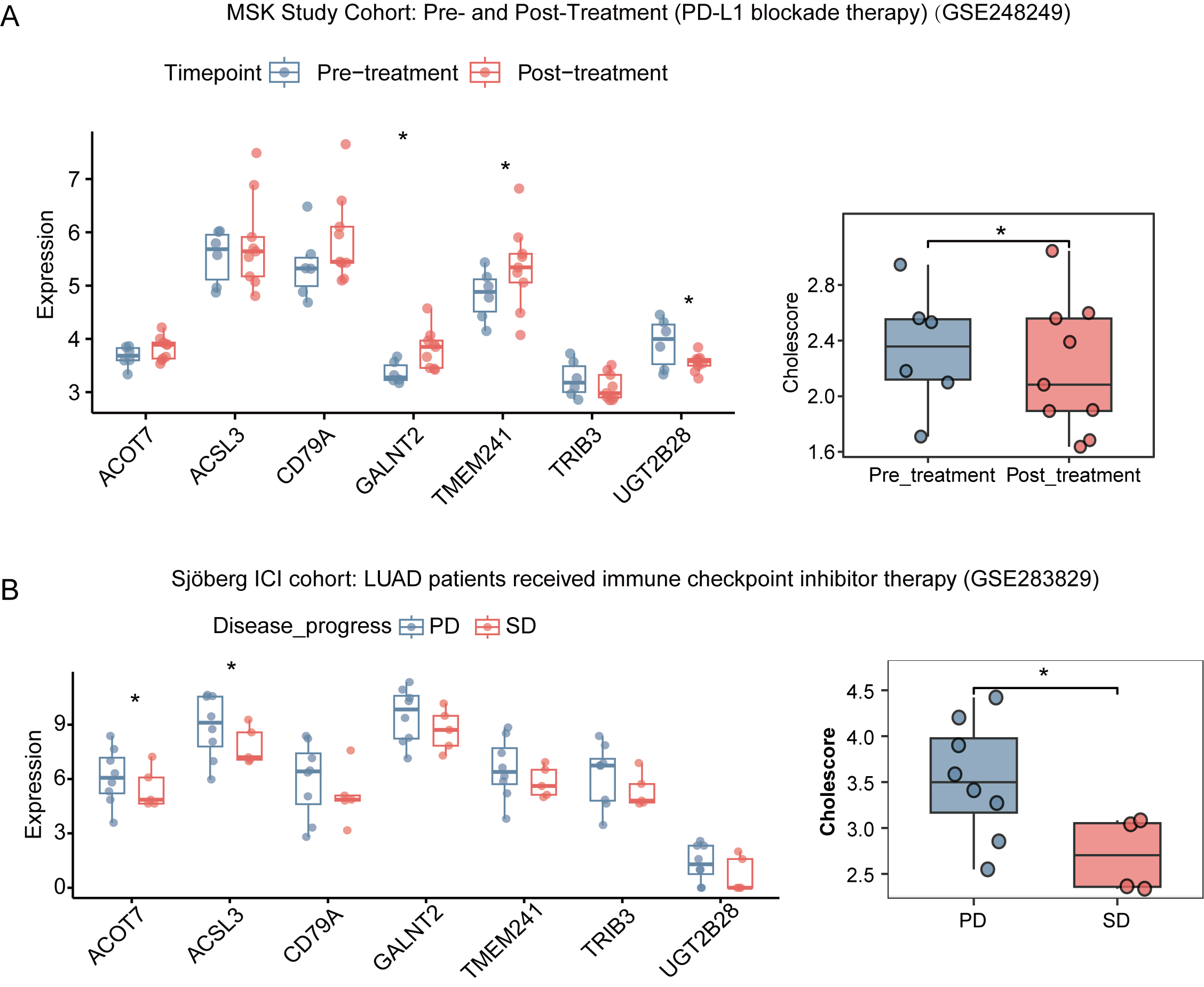
**

**Figure S3** Survival analysis of Estrogenscore and LUAD overall survival in different subgroup patients. Differential expression scatter plot and K-M curves of Estrogenscore and LUAD overall survival in different gender (A), age at diagnosis (B), T stage (C), N stage (D) and pathologic stage (E) patients. NS = no significant, *p< 0.05, **p< 0.01, and ***p< 0.001.


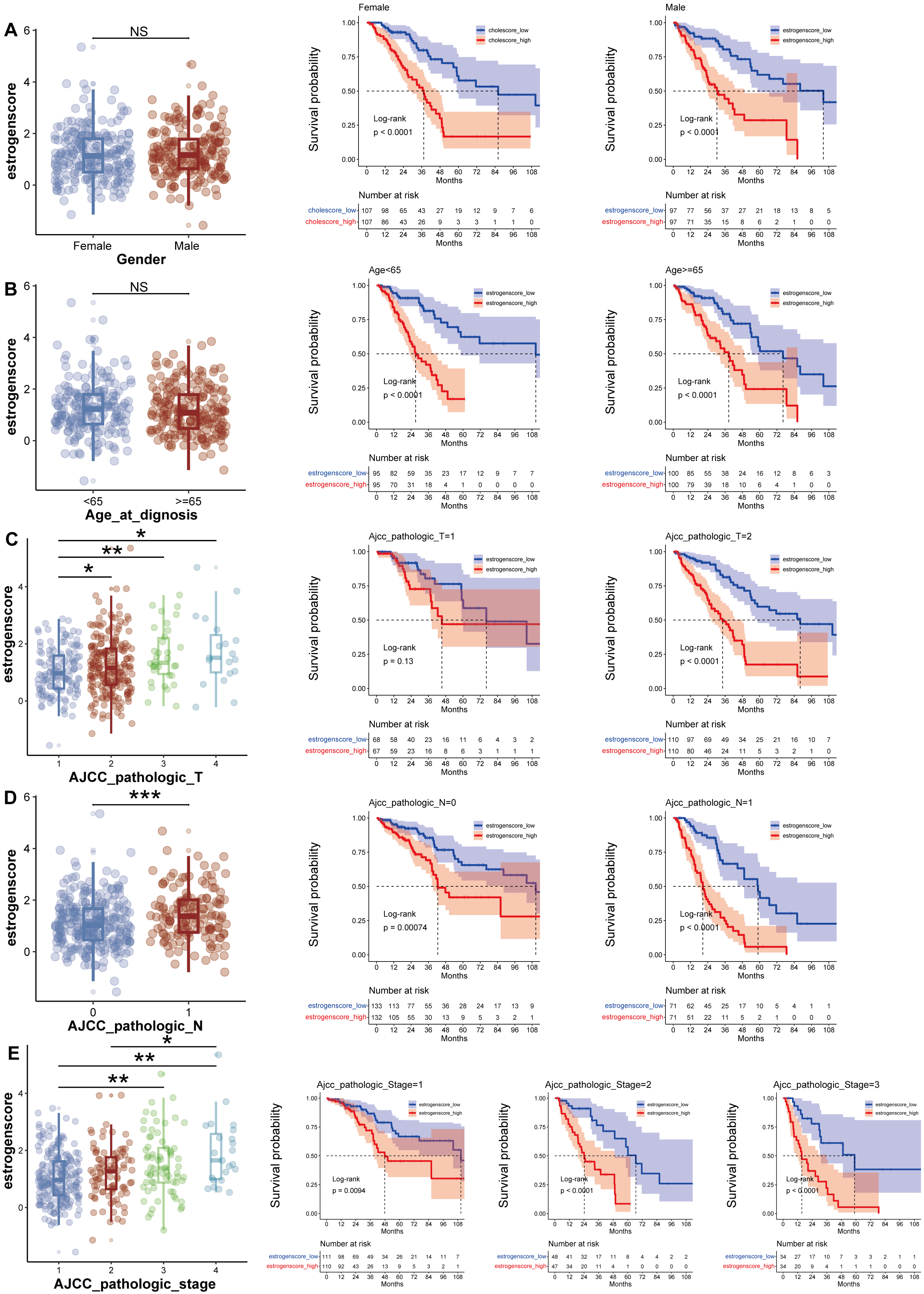


**Figure S4** The correlation between risk scores and overall survival of LUAD patients in different scores’ subgroups after stratification by gender (A), T-stage (B) and clinical stage (C).


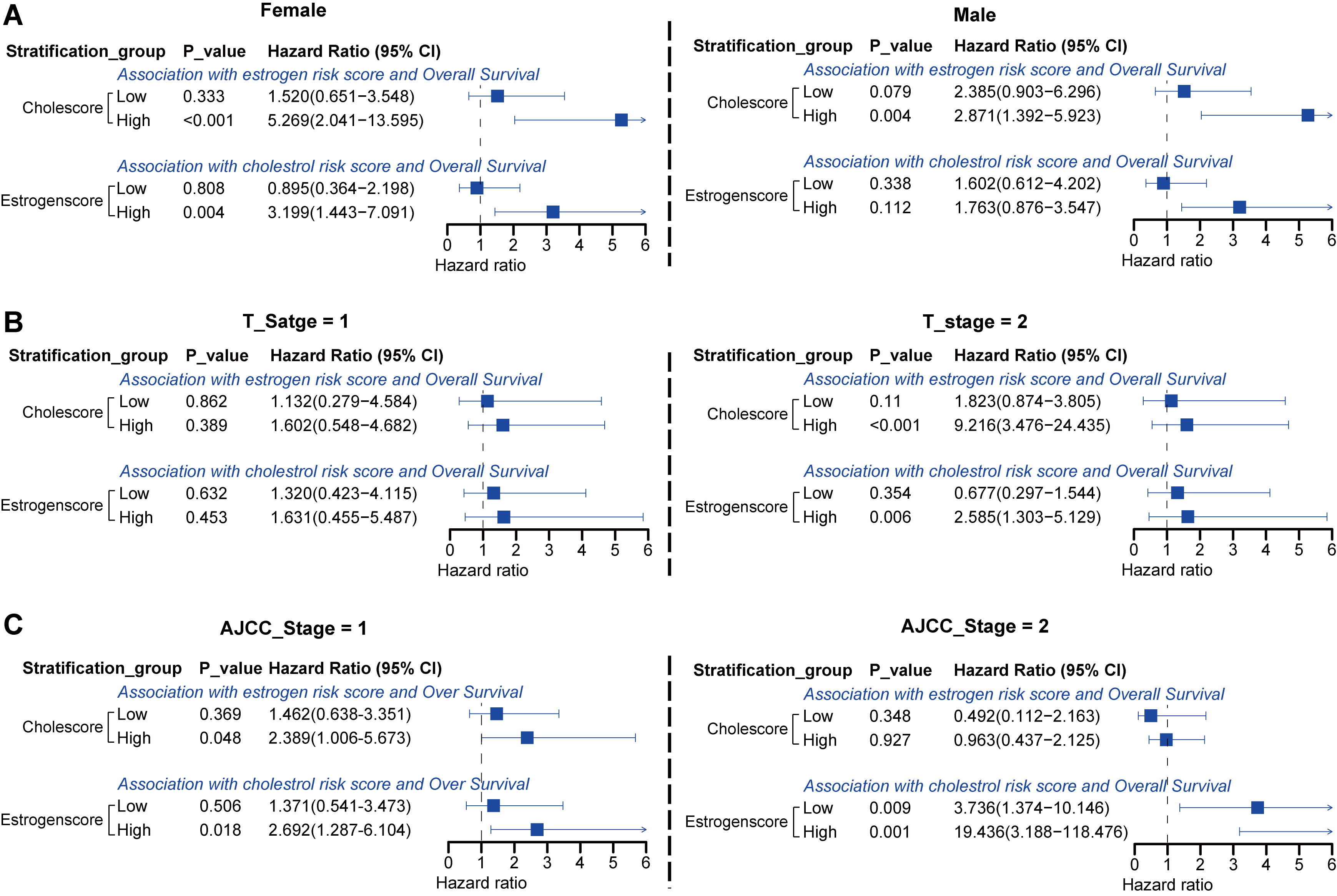


**Figure S5** The correlation between overall survival and clinical pathological parameters, risk scores, and interaction terms in male and different T-stagepatients with lung adenocarcinoma.


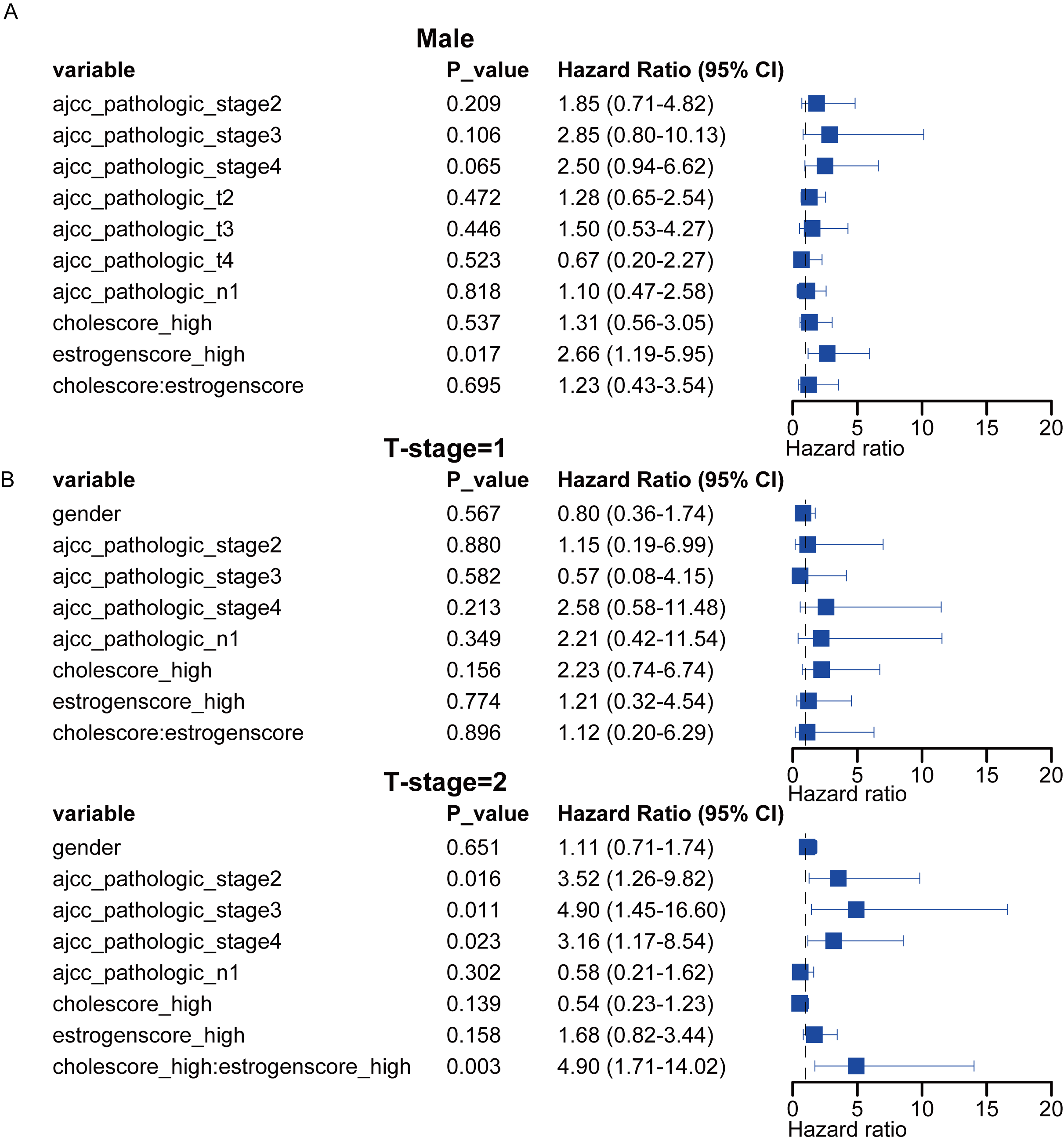


**Figure S6** Joint associations of Cholescore and Estrogenscore and overall survival of lung adenocarcinoma in different gender (A), T stage (B) and clinical stage (C) patients.


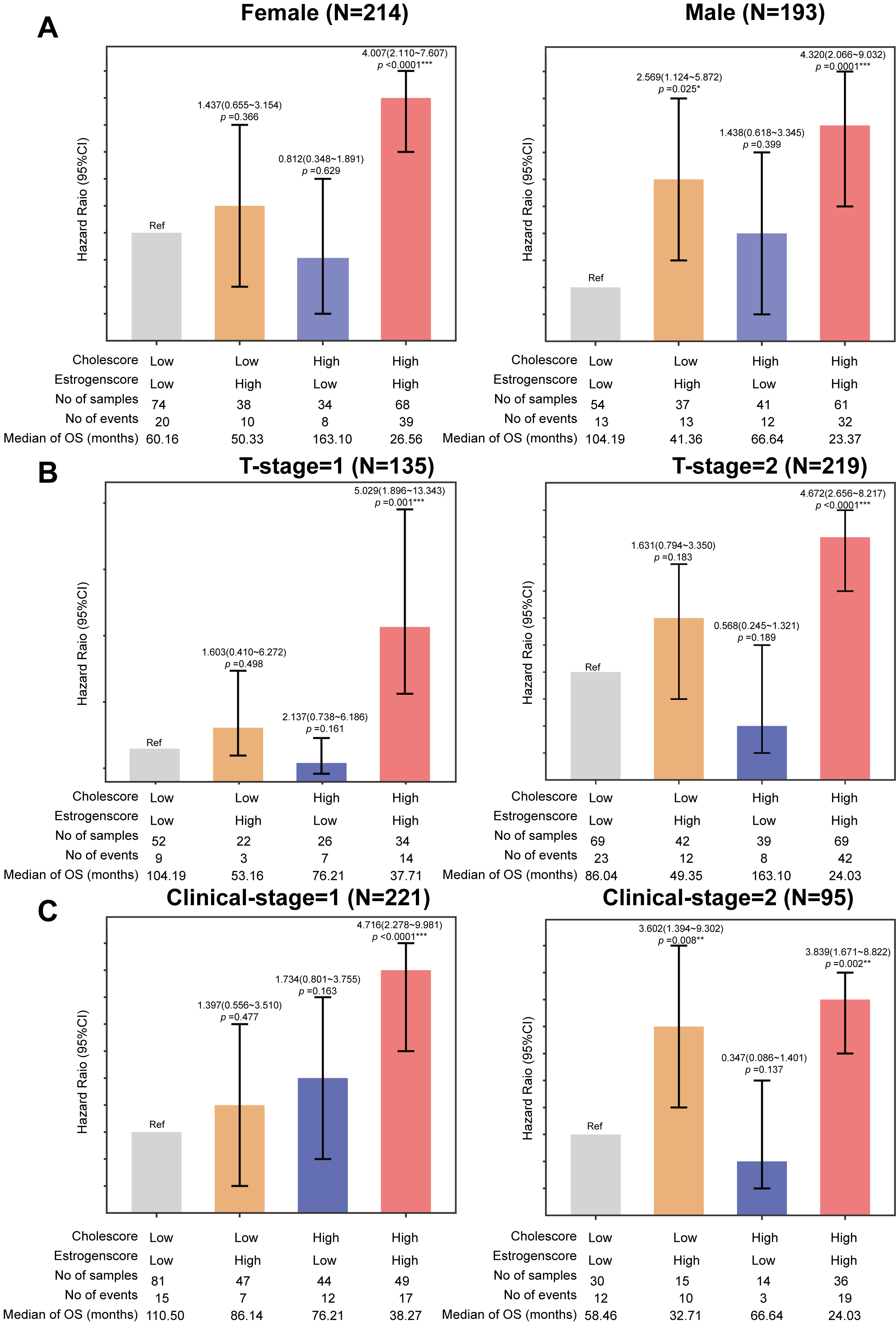


**Figure S6** The results of the optimization analysis of the cutoff values for Cholescore (A~C)and Estrogenscore(D~F) (including the median, quartiles, and the optimal cutoff values based on the data).


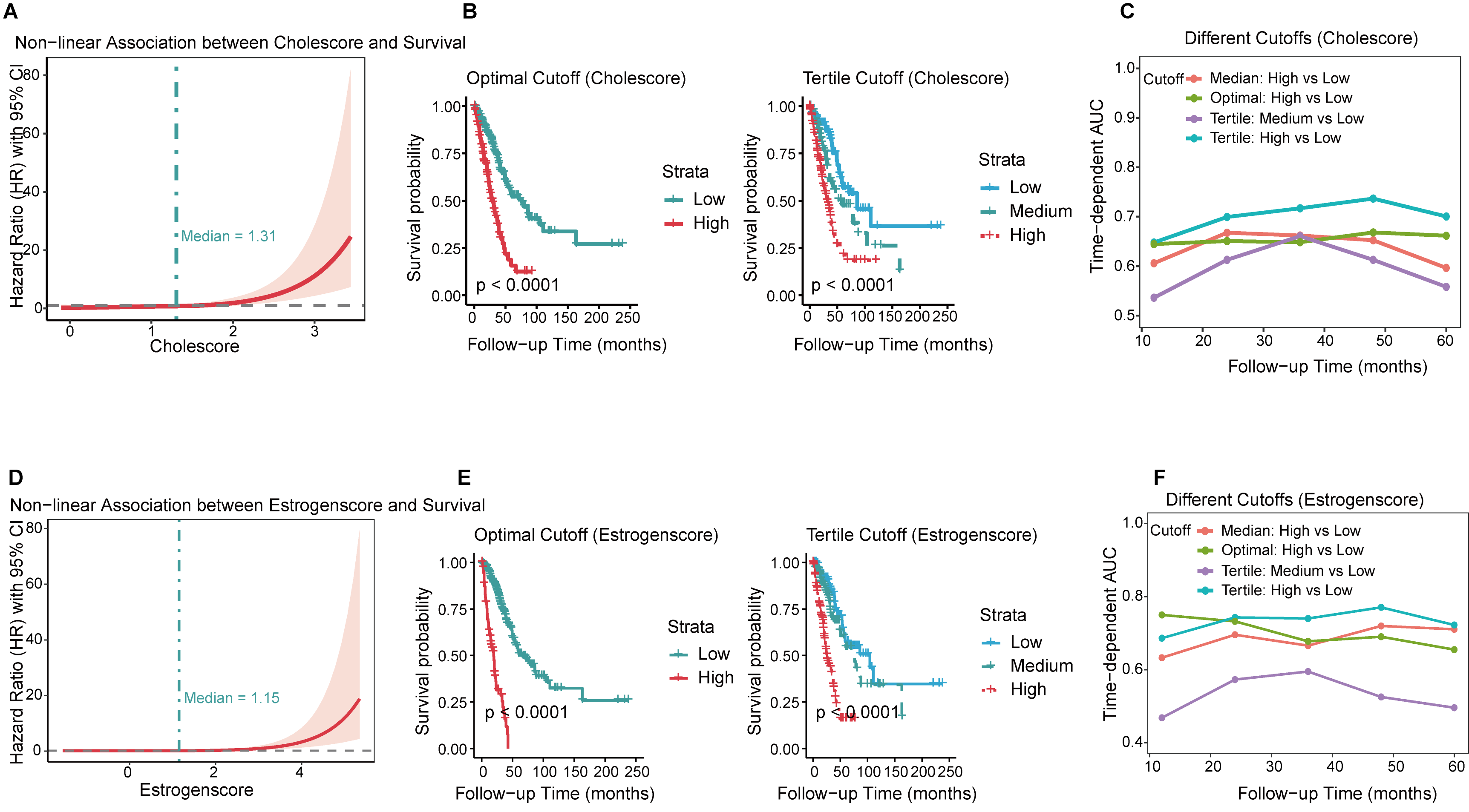

Supplement: Supplementary file 1 — Supplementary Material 1 [file 41598_2025_22140_MOESM1_ESM.docx]
